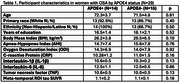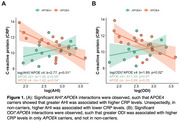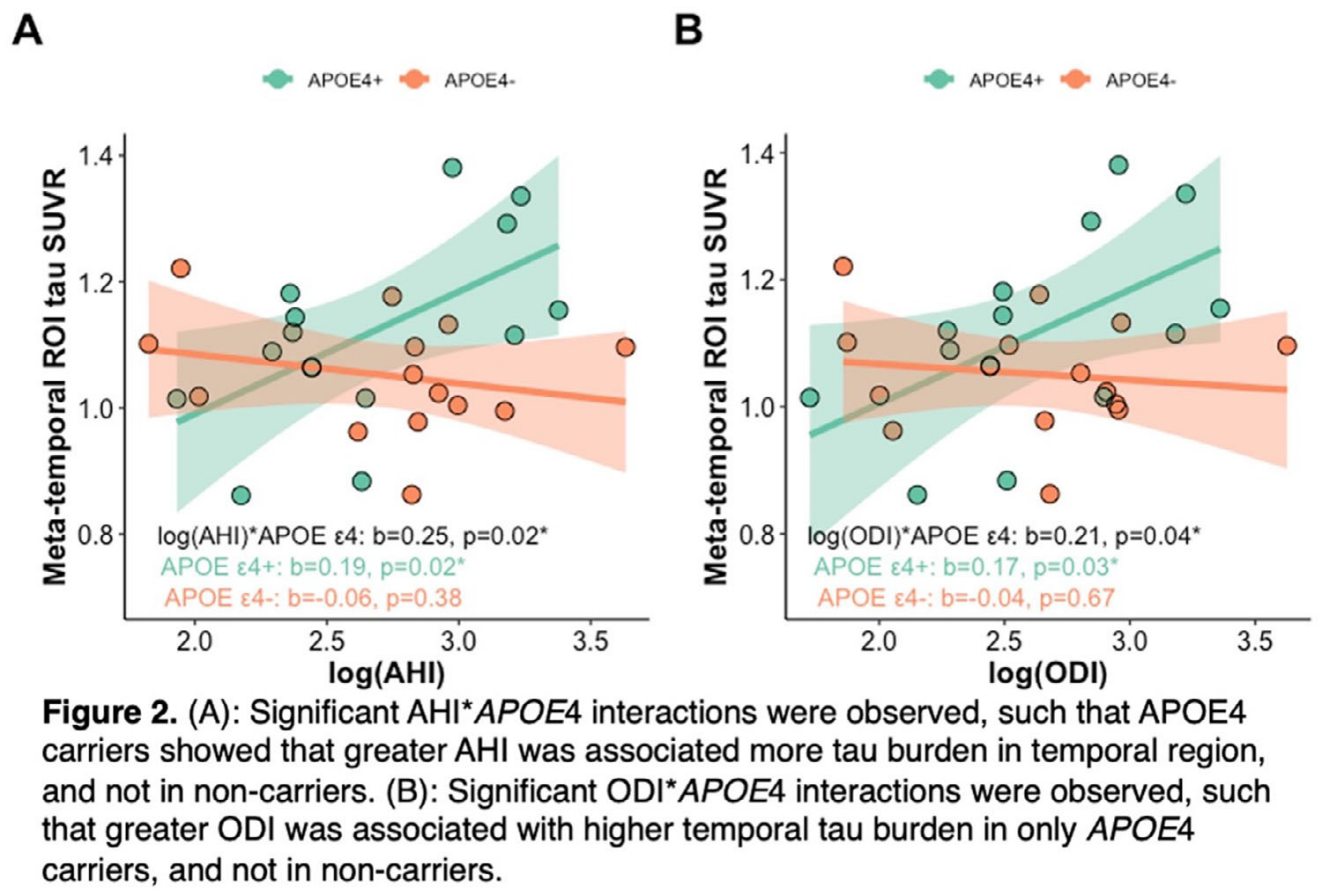# *APOE*4 modifies the association between sleep apnea, inflammation, and tau pathology in older women

**DOI:** 10.1002/alz70862_109805

**Published:** 2025-12-23

**Authors:** Kitty K. Lui, Xin Wang, Ella T Lifset, Nadine C. Heyworth, Breanna M Holloway, Pamela N DeYoung, Atul Malhotra, Erin E. Sundermann, Sarah J Banks

**Affiliations:** ^1^ SDSU / UC San Diego Joint Doctoral Program in Clinical Psychology, San Diego, CA USA; ^2^ University of California, San Diego, La Jolla, CA USA; ^3^ UC San Diego, La Jolla, CA USA; ^4^ UC San Diego Health, La Jolla, CA USA

## Abstract

**Background:**

Obstructive sleep apnea (OSA) is thought to elevate Alzheimer’s disease (AD) risk, possibly through an inflammatory mechanism. This may be particularly relevant for older women, who are underdiagnosed for OSA, express higher inflammatory responses and accumulate greater pathological tau in early AD stages than older men. *APOE*4 seems to worsen OSA’s impact on AD risk; however, findings are mixed. This study examined *APOE*4’s moderating role in how OSA symptoms relate to inflammation and tau pathology among older women with OSA from the Women: Inflammation and Tau Study (WITS).

**Method:**

WITS recruits older women with mild cognitive impairment on the telephone Montreal Cognitive Assessment and elevated AD polygenic hazard scores and/or family history of dementia. Participants included 43 women (aged:71.9±4.1 years) with cerebrospinal fluid‐derived inflammatory markers (IL‐1β, IL‐6, and TNF), MK‐6240 tau PET data from a composite meta‐temporal region of interest and the following OSA characteristics from a home sleep test: apnea‐hypopnea index (AHI) and oxygen desaturation index (ODI). Following clinical guidelines, OSA was diagnosed with AHI≥5/hr. In women with OSA, multiple regression models analyzed OSA characteristics×*APOE4* interactions on inflammatory markers and tau standardized uptake value ratio, controlling for age and body mass index (BMI).

**Result:**

29 women met OSA diagnosis (67%) with 27 unaware of this at screening (Table 1). In women with OSA, AHI and ODI significantly interacted with *APOE*4 to predict CRP (*p*s<0.04), whereby greater OSA severity related to elevated CRP levels in *APOE*4 carriers (AHI: *p*<0.05; ODI: *p* = 0.07). However, in non‐carriers, higher AHI was associated with lower CRP (*p* = 0.04), while ODI was not associated with CRP (*p* = 0.24; Figure 1). Further, AHI and ODI significantly interacted with *APOE4* status to predict tau (*p*s<0.04), such that greater OSA severity related to higher tau burden in only *APOE*4 carriers (*p*s<0.04) (Figure 2). There were no significant OSA severity×*APOE*4 interactions nor main effects of OSA severity on other inflammatory markers.

**Conclusion:**

In women with OSA, *APOE*4 interacts with OSA severity to potentially precipitate a stronger inflammatory response and higher tau burden. Early diagnosis and treatment of OSA might mitigate AD risk, especially for older female *APOE*4 carriers.